# Mesoporous Silica Nanomaterials: Versatile Nanocarriers for Cancer Theranostics and Drug and Gene Delivery

**DOI:** 10.3390/pharmaceutics11020077

**Published:** 2019-02-13

**Authors:** Samuel Kesse, Kofi Oti Boakye-Yiadom, Belynda Owoya Ochete, Yaw Opoku-Damoah, Fahad Akhtar, Mensura Sied Filli, Muhammad Asim Farooq, Md Aquib, Bazezy Joelle Maviah Mily, Ghulam Murtaza, Bo Wang

**Affiliations:** 1 Department of Pharmaceutics, School of Pharmacy, China Pharmaceutical University, Nanjing 211198, China; kessejnr@yahoo.com (S.K.); otiboakye1000@gmail.com (K.O.B.-Y.); Biasfili@yahoo.com (M.S.F.); pharma1154@yahoo.com (M.A.F.); mdaquib0007@yahoo.com (M.A.); mmaviah@yahoo.fr (B.J.M.M.); 2 School of Basic Medicine and Clinical Pharmacy, China Pharmaceutical University, Nanjing 211198, China; belyndaochete@yahoo.com; 3 Australian Institute for Bioengineering & Nanotechnology, The University of Queensland, St Lucia, Brisbane, QLD 4072, Australia; yawdamoah@gmail.com; 4 School of Life Science and Technology, China Pharmaceutical University, Nanjing 210009, China; fahadakhtar121@outlook.com; 5 Department of Pharmacy, COMSATS University Islamabad, Lahore Campus 54600, Pakistan; gmdogar356@gmail.com

**Keywords:** cancer theranostics, mesoporous silica nanomaterials, gene therapy, drug delivery, stem cell, cancer therapy

## Abstract

Mesoporous silica nanomaterials (MSNs) have made remarkable achievements and are being thought of by researchers as materials that can be used to effect great change in cancer therapies, gene delivery, and drug delivery because of their optically transparent properties, flexible size, functional surface, low toxicity profile, and very good drug loading competence. Mesoporous silica nanoparticles (MSNPs) show a very high loading capacity for therapeutic agents. It is well known that cancer is one of the most severe known medical conditions, characterized by cells that grow and spread rapidly. Thus, curtailing cancer is one of the greatest current challenges for scientists. Nanotechnology is an evolving field of study, encompassing medicine, engineering, and science, and it has evolved over the years with respect to cancer therapy. This review outlines the applications of mesoporous nanomaterials in the field of cancer theranostics, as well as drug and gene delivery. MSNs employed as therapeutic agents, as well as their importance and future prospects in the ensuing generation of cancer theranostics and drug and therapeutic gene delivery, are discussed herein. Thus, the use of mesoporous silica nanomaterials can be seen as using one stone to kill three birds.

## 1. Introduction

Irregular and uncontrollable cell growth is highly associated with cancer. With metastasis and invasion associated with malignant phenotypic behavior, cancer aggressively attacks diverse areas of the human body and is widely believed to be one of the most long-lasting ailments worldwide [1,2]. Over the past decade, the incidence and death rate associated with cancer has risen sharply. According to the 2015 World Health Organization figures, cancer is one of the chief causes of death in industrialized nations. In unindustrialized nations, it is second only to cardiovascular disease as the main cause of fatality. It is expected that by 2020, the number of deaths from cancer will comprise 13% of the total deaths worldwide [3,4]. Because cancer is characterized by different stages and a given patient’s age and health pedigree, the management of cancer must be personalized and combined with quite a few other therapies. Current malignant cell treatment models, such as chemotherapy, surgery, photodynamic therapy (PDT), and radiotherapy, are capable of prolonging a patient’s life to some degree and helping them to live longer. However, radiotherapy has detrimental effects, such as the risk of minor malignancy at the exposed zone, and it can go so far as to damage live and strong tissues. Chemotherapy is characterized by the use of a variety of chemotherapeutic agents to kill cancer cells and stop its proliferation. A number of chemotherapeutic agents are not cell-specific and, thus, are capable of destroying normal cells and further contributing to permanent systemic problems. In addition to not being cell-specific, the nuisance of multidrug resistance (MDR), exhibited by most cancer cells, poses as a serious limitation and contributes to the low therapeutic index (TI) of chemotherapy [5]. Other systems, such as photodynamic therapy (PDT), which employ agents that are photosensitizing in nature to destroy cancer cells, have also been proposed. In photodynamic therapy, the agents are operative the moment they are triggered via a definite form of light; this light should be specifically geared towards the cells, making photodynamic therapy (PDT) extra specific and not as toxic as chemotherapy [6]. The restrictive aspect of this technique, conversely, is the inability of light to reach deep-sighted tumors within the body, as well as the change in the multidrug resistance toward the photodynamic therapy agents present in the cancer cells that have been treated [7]. As a result of the limitations of these treatments, metastasis, the recurrence of the tumor, resistance to the therapy being performed, and adverse effects triggered as a result of chemotherapy and radiotherapy persist as the key setbacks in cancer therapy. Thus, orchestrated approaches are immediately required in order to effectively treat cancer. One promising, emerging discipline is nanomedicine, which encompasses nanotechnology and biomedicine. Nanomedicine has great potential for expanding the treatment of various ailments, including diabetes, tissue engineering, and heart ailments, as well as great potential applications in cancer theranostics. The rapid emergence of novel nanomaterials has become a great platform for overcoming the adverse effects of chemotherapy, even without the earliest stage diagnosis of cancer. Doxil, as the foremost Food and Drug Administration (FDA)-accepted nanodrug, is a distinctive example, in which Doxorubicin (DOX) stays encapsulated in a liposome complex to ensure sustained flow time and bioavailability of the drug, as well as minimal adverse effects towards cardiac muscles and other body tissues [8]. As another example, in 2011, Cornell dots (C-dots) were accepted by the Food and Drug Administration (FDA) for a stage-I human clinical trial, thus becoming the earliest silica-based cancer indicative nanoparticles. C-dots are dye-encapsulated silica nanoparticles of a very minute size (less than 10 nm), which can be used as a tool to help surgeons to detect tumors [9]. Thus, remarkable approaches have been taken to fabricate nanomaterials for cancer theranostics. Bio-imaging coupled with cancer therapy has been demonstrated by Loo et al., who fabricated nanoshells targeted at the immune system to identify and kill breast cancer cells [10]. Other studies have also made immense breakthroughs [11]. In the same way, mesoporous silica nanomaterials (MSNs) possess great potential as functionalized nanoparticles. An early example of their utility involved a process in which folic acid (FA) was synthesized, and modified MSNs were used for targeted transfer of the water-resistant anticancer medication camptothecin [12,13]. In totality, the research performed has revealed that (1) mesoporous silica nanoplexes have substantial in vitro and in vivo cancer destruction capabilities, and (2) they have applications in imaging and cancer therapy simultaneously [12,14]. As research into nanomaterials continues to emerge, nanomedicine as a field of study is predicted to serve an important role in cancer diagnosis and treatment. Silica is one of the peak natural resources available on earth and plays a crucial role in medicine, mainly with respect to human skin and bones and so on [15]. Categorized by the FDA as generally recognized as safe (GRAS), silicon dioxide is generally employed as a food additive and in the cosmetics and pharmaceutical industries. Because of the role of silica in assuaging biosafety concerns and the ease of the processes involved in the fabrication of silica, silica nanomaterials play a very vital role in biomedical studies. Over the last decade, mesoporous nanomaterials have attracted growing attention in the fields of optical imaging, magnetic resonance imaging (MRI), photodynamic therapy, and drug delivery [16,17,18,19,20,21,22]. After MCM-41-type MSNs were discovered as nanovehicles that could be used to convey drugs in 2001 [22], different types of mesoporous materials, such as TUD-1 [23], SBA-15 [24,25], MCM-48 [26], HMM-33 [25], and FSM-16 [27], have been fabricated and used for the delivery of drugs. In the field of drug delivery, they have numerous benefits: (i) Varying particle size (50–300 nm) allowing superficial endocytosis with marginal cytotoxic effects; (ii) large pore volume and internal surface area, thus making MSNs good delivery vehicles for drugs; (iii) a firm matrix structure enabling MSNs to be evenly distributed in water and repel modifications as a result of heat, pH, and hydrolysis-induced breakdown, as well as mechanical stress; (iv) narrow aperture as a result of their tunable, porous structure, which allows the loading of diverse therapeutic agents possessing drug release kinetics that are specific; (v) their use in targeted delivery and controlled release because their internal and external surfaces can be selectively functionalized; and (vi) a distinctive, porous configuration, inhibiting the untimely release of the loaded constituents while their pores are not completely covered [20,21,28,29,30]. MSNs show more wide-ranging prospects than other drug delivery systems and offer encouraging grounds for concurrent cancer diagnosis and therapy, as well as drug and gene delivery. This review outlines the current state of the research of using MSNs as drug vehicles in the field of cancer theranostics and gives a summary of the current research breakthroughs and imminent advancements in using MSNs as a biomedical agent, principally paying attention to the hands-on uses of MSNs as carriers for anticancer agents, poorly soluble drugs, and therapeutic genes. This concise review paper moreover highlights the up-to-date advancements in the field of nucleic acid delivery using MSNs, specifically for the conveyance of pDNA and siRNA and the combinatorial delivery of nucleic acids and drugs.

## 2. Cancer Therapy Using Silica Nanomaterials

### 2.1. Detection and Diagnosis of Cancer

In the past few years, numerous far-reaching studies have been geared towards effective and harmless treatment of cancer as well as numerous reviews written to cover mesoporous silica nanomaterials as drug delivery vehicles [31]. The arrival of nanomaterials serves as a great platform that can be used for early detection and diagnosis of cancer. Silica-based nanomaterials play an important part in the treatment of cancer because of their discrete advantages—for example, interior pore volume plus high surface area, tunable and even pore size, nontoxicity, and biocompatibility. For these reasons, they have several advantages (Table 1). 

In order to overcome the hurdle and provide appropriate and extra real treatment of cancer, early detection and diagnosis are vital in preventing death. As it stands, the process where tissues are taken from the patient to search for cancerous cells—i.e., tissue biopsy—stands as the most extensively used means of identification. From time immemorial, a number of nanomaterials, for example, quantum dots (QDs) [36], gold nanomaterials [37], then silica nanomaterials [38], were employed for initial cancer detection and diagnosis. Though gold nanomaterials have so far attained positive improvement in the treatment of cancer, using optical imaging with gold nanomaterials as the agent has a narrow clinical future [39]. Quantum dots (QDs) possibly remain as multipurpose nanomaterials used in the biomedical field with in situ optical imaging and drug delivery, but much work has to be geared towards their toxicity effects before their widespread use in medical diagnosis and therapy [40]. By contrast, silica nanoparticles have no toxicity, are biocompatible, biodegradable, and they possess extraordinary loading competency for different agents, which in effect makes them ideal candidates for harmless and effective therapy.

### 2.2. Imaging Contrast Agents Using Silica Nanomaterials

**Figure 1:**
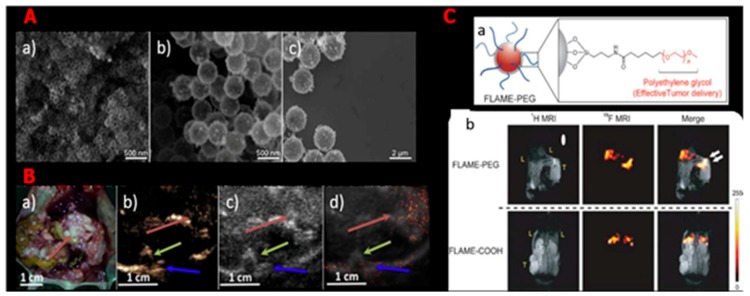
Silica nanoparticles used as contrast agents for ultrasound and magnetic resonance imaging. (**A**) SEM images of porous hollow silica nano- and microshells having different particle size: (**a**) 100 nm, (**b**) 500 nm, and (**c**) 2000 nm. (Reproduced from Reference [32] with the permission of Elsevier Ltd.) (**B**) Ultrasound imaging of gas-filled silica microshells present in tumor-bearing mice; (**a**) intraperitoneal IGROV-1 ovarian tumor in the dissected nu/nu mouse, (**b**) Cadence contrast pulse sequencing (CPS) image, and (**c**) B-mode image of the particles through the tumor cross-section one (1) hour post injection with silica nanoparticles, (**d**) overlay of CPS image and B-mode image (red arrow = tumor, green arrow = spinal column, blue arrow = bottom of the mouse). (**C**) In vivo accumulation of silica nanoparticles at tumor site; (**a**) structural diagram of functionalized silica nanoparticles, (**b**) in vivo MRI of functionalized silica nanoparticles in tumor-bearing mice. Liver and tumor sites are indicated as L and T, respectively. Reproduced from Reference [33] with the permission of Wiley-VCH Verlag GmbH & Co. KGaA, Weinheim.

Ultrasound (US) as well as magnetic resonance imaging (MRI) are mainly employed as a tool for diagnosis of cancer as they are economical, have real-time monitoring characteristics, and possess low radioactivity [38]. Nonetheless, the generally employed agents for MRI or US are minute molecules comprising calcium and gadolinium chelates as well as metallic ions [41], which have the disadvantage of not being able to deliver high contrast images that can aid in diagnosing cancer early [38]. Because they possess a high drug loading capability, high strength, and can easily degrade in the body in an apt way, silica nanomaterials are employed as US and MRI contrast agents with target specificity and low toxicity and demonstrate future prospects for cancer diagnosis (Figure 1A–C). Perfluorocarbon gas-filled hollow porous silica micro shells were engineered to be directly injected into tissues in 2010. These nanomaterials are capable of remaining in the tissues for a number of days deprived of lethal effects. Because these agents were able to stay in the body for long, it was perceived that they could be applied in novel applications, such as Doppler imaging via ultrasound as well as contrast specific imaging, for a lengthier time [42]. Recently, hollow mesoporous silica nanomaterials (HMSNs), as well as silica–boron nanomaterials, were used in mice for ultrasound (US) imaging [32]. For instance, Daewon et al. fabricated MSNs with Herceptin, an agent that can precisely target definite forms of breast cancers. These engineered MSNs were capable of conferring a sufficiently average pixel strength to create higher-quality US images [43]. MRI is capable of penetrating underlying tissues and delivering important high spatial resolution data void of radiation; nevertheless, the aforementioned needs extremely penetrating contrast agents for concrete use [44]. Advanced research works have studied the chance of employing silica nanomaterials as a unique form of MRI agent [45]. The in vivo sensitivity of MSNs as well as other MRI nanomaterial probes have similarly been matched. A prostate tumor-bearing mouse was injected with hyperpolarized silica nanoparticles and may well be easily imaged with high sensitivity at low magnetic field, successively displaying additional uses, such as real-time MRI [46]. Additionally, Kazuya et al. fabricated a novel MRI contrast agent comprising a core micelle with liquid perfluorocarbon engulfed inside a vigorous silica shell [33]. This kind of silica nanomaterial-based agent possesses satisfactory in vivo stability, high sensitivity, a surface that can be modified, and biocompatibility, which has future prospects in timely cancer discovery and treatment. 

### 2.3. Silica Chips Mesoporous Nanomaterials

**Figure 2:**
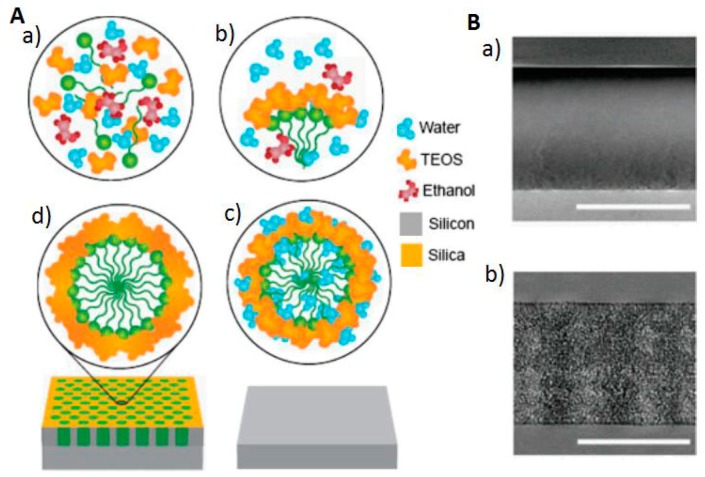
Production and assembly of mesoporous silica-based chips for proteomic uses. (**A**) Chemical changes taking place in the solution during the production phases of a mesoporous silica film; (**a**) fresh solution, (**b**) Micelles formation, (**c**) spin-coating process leading to self-assembly, (**d**) magnified image of a pore post-aging at high temperature. (**B**) SEM and TEM cross-sectional images of GX6 chip on a (**a**) bulk silicon wafer surface (upper) and (**b**) mesoporous silica film-coated silicon wafer surface (lower). Scale bar is 500 nm. Reproduced from Reference [34] with the permission of Wiley-VCH Verlag GmbH & Co. KGaA, Weinheim.

MS and CT serving as means of proteomic analysis have transformed the early detection of cancer and are capable of exhibiting the dissimilarities between regular cells and cancer cells [47,48,49]. Yet, numerous limitations need to be addressed [50,51]—for instance, the signal interference when moving from high to low concentrations of proteins, individual spectra properties of CT or MS, and bad selectivity to detect changes in protein concentration amid the typical and atypical forms. Furthermore, the old models have multifaceted preparation methods. These complications render detection inaccurate and slow [52,53]. Silica-based chips (mesoporous in nature) (Figure 2A,B) with precise inert properties and pore size serve as a filtration tool in protein MS to help identify cancer biomarkers [34,54,55,56]. Nanoporous silica chips permit the partitioning of high molecular weight protein from those that have a low molecular weight [56]. As a result, signaling is enhanced because low molecular weight proteins become more concentrated. Further, surface engineering through the addition of metallic ions [54] and extra functional groups [57] boosts the sensitivity and selectivity of silica chips that are mesoporous in nature and capable of isolating proteins with low molecular weight and identifying biomarkers cancers. Functionalized mesoporous silica chips hence offer a hopeful mark for the potential diagnosis of early symptoms of cancer and other diseases.

## 3. Comprehensive Cancer Theranostics Using a Tri-Functionalized Silica Nanomaterial

**Figure 3:**
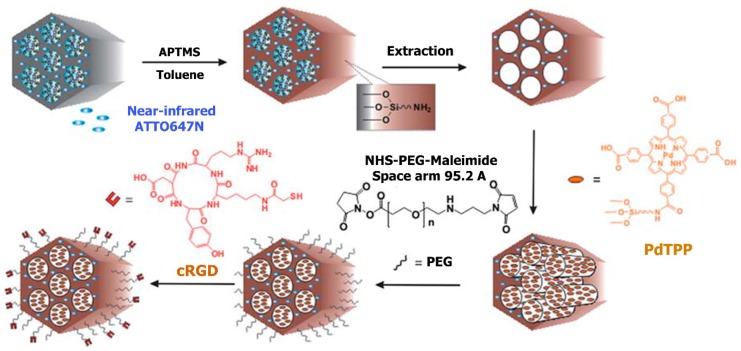
Synthesis of the tri-functionalized mesoporous silica nanomaterial (MSN), A647@MSN-RGD-PdTPP. The nanomaterials were initially functionalized with ATTO 647N, then photodynamic therapy photosensitizer (PS) agent (APTMS-PdTPP), and lastly with the tumor targeting ligand (cRGD). Reproduced from Reference [58] with permission from The Royal Society of Chemistry.

**Figure 4:**
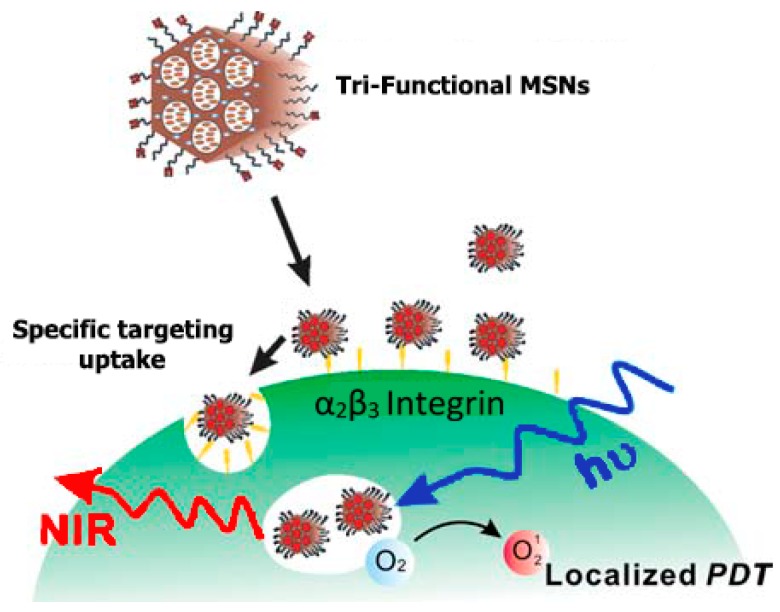
Tri-functionalized mesoporous nanomaterials employed as theranostic compounds that possess distinctive domains for localized photodynamic therapy photosensitizers, traceable imaging, as well as capable targeted delivery. Reproduced from Reference [58] with permission from The Royal Society of Chemistry.

Shih-Hsun et al. [58] reported in their work an amazing discovery of the fabrication of the tri-functionalized mesoporous silica nanoparticles (MSNs) that can be used in the field of theranostics to coordinate the trio of target, imaging, and therapy in a distinct unit (Figure 3). Mesoporous silica nanoparticles (MSNs) were functionalized with contrast agents that permit imaging of particle targeting to be traced, drug cargos used as therapeutic mediators, and biomolecular ligands for highly-targeted particle carriage. For traceability, ATTO647N, which is a near-infrared (NIR) fluorescent contrast agent, was directly incorporated into the silica structure of the nanomaterial, in order to know the transparency of most tissues at near-infrared (NIR) wavelengths and increase the nanomaterials surface area accessible for the successive targeting ligands and conjugating drugs. Pd-porphyrin (PdTPP), which is an oxygen-sensing, palladium-porphyrin based photosensitizer, was fused in the mesoporous nanomaterials’ nanochannels in order to allow photodynamic therapy (PDT). cRGDyK peptides, found on the surface of the mesoporous nanomaterials, were employed for targeting the overexpressed avb3 integrins of cancer cells, and to guarantee the internalization of the photosensitizer PdTPP (Figure 4). Evaluation of the cells in vitro of the theranostic area was carried out, and it revealed not just outstanding specific targeting and negligible impairment but a very compelling therapeutic effect, too. Photodynamic therapy is a novel therapeutic technique for the treatment of cancer, which uses light of appropriate wavelength and a targeted photosensitizer (PS). These modules are brought together to cause cellular or tissue effects that depend on oxygen [59]. Considering PDT, light induces death or damage of the cells by generating free radicals and reactive oxygen (e.g., ^1^O_2_). Huge volumes of photosensitizer (PS) can be incorporated in mesoporous silica nanomaterial canals, and the outer layer of the nanomaterials can be chemically modified for targeting MSNs or increasing their cellular uptake [60]. The momentary ^1^O_2_ must directly react with molecules in intracellular pathways to yield a maximum cytotoxic effect, and the distinctive framework of the nanomaterials can shield PS from degrading environs. A thoroughly examined photosensitizer (PS), protoporphyrin IX, was taken up by cells well and elicited phototoxicity, which relied on the irradiation time and light intensity. The principal advantage of photodynamic therapy in cancer treatment is the fact that cytotoxicity transpires only in areas experiencing the radiation, and any cells lying outside these areas are not compromised. Hsia et al. successfully demonstrated in 2011 that 2-photon activated-photodynamic therapy (TPA-PDT) permitted spatially selective treatment of cancers as well as enhanced the depth to incident light which penetrated tissues. MSNs co-encapsulating 2-photon-absorption dyes and PS boosted the high-energy transfer frequency for TPA-PDT. The well-organized mesoporous framework of silica nanomaterials caused a surge in the energy transfer rate as high as 93%. The cytotoxicity induced by the ^1^O_2_, as a result of intracellular energy transfer, was also very much competent in vitro and in vivo in breast cancer prototypes [61]. These outcomes propose that mesoporous silica nanomaterials modified for TPA-PDT are capable of being used in clinical settings. 

## 4. Mesoporous Silica Nanomaterials in Theranostic Stem Cell Therapy

**Figure 5:**
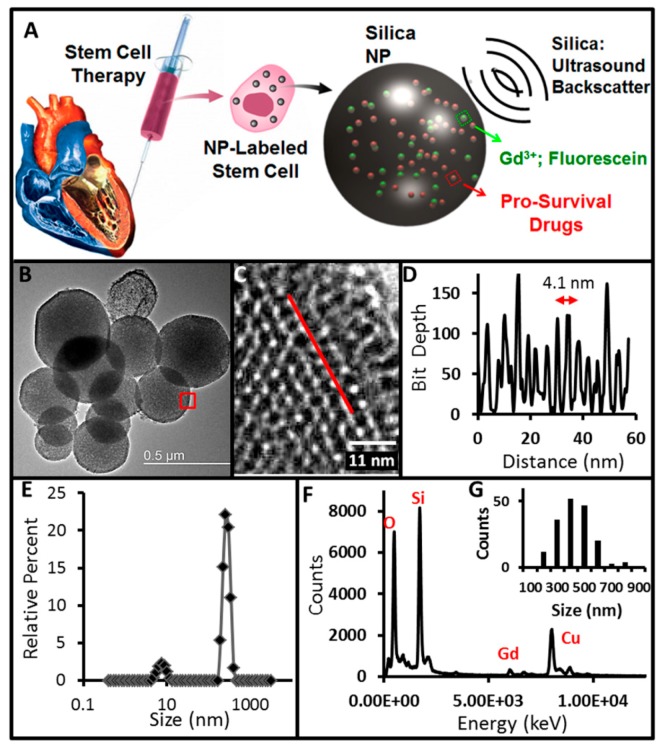
(**A**) MSNs possess impedance disparity to backscatter ultrasound, MRI signal via Gd^3+^, and an optical signal from fluorescein. The same nanoparticles increase MSC survival by ensuring insulin-like growth factor (IGF) sustained release. (**B**) TEM images illustrate mesoporous particles with 4.1 nm pores (**C**) determined by line profiling (**D**). The red box present in (**B**) shows the area imaged at a higher magnification in (**C**). The line in C is representative of the profile used to create (**D**). (**E**) DLS data of the MSNs. Additional characterization by EDS shows projected peaks for silicon and oxygen along with gadolinium from the secondary tag (**F**). The copper signal in (F) is from the TEM grid. Panel (**G**) is a histogram of MSN sizes from the TEM data in nanometer (nm). Reproduced from Reference [91] with permission from the theranostics ivyspring international publisher.

In the treatment of heart diseases, cardiac stem cell therapy has given rise to high hopes [62,63,64] even though, hitherto, there has been the major setback of poor long-standing efficacy owing to fast cell death when implantation is carried out [63]. This problem is due to two basic points: (i) Implanting into extremely fibrotic tissue [65,66] and (ii) the treated region being ischemic or inflamed [67]. A very small amount of implanted stem cells does not die as a result of these barriers, after 4–8 weeks [66], when cells are mis-injected in a larger number of patients [68]. In order to combat any ischemic and inflammatory conditions, prosurvival blends have proven vital to improving stem cell growth [69], but a big limitation is the short half-life and poor bioavailability of these agents. Implantation into fibrotic tissue can be avoided using echocardiography, but the lack of available contrast agents limits cellular imaging for surgical guidance. Ultrasound (US) is one of the methods for real-time imaging of cell delivery as a result of its many centimeters of imaging depth and high spatial/temporal resolution. US is already regularly used in echocardiography and in the delivery of stem cells. Contrast agents for US molecular signaling are principally made of perfluorocarbons—i.e., liquid droplets [70] and gaseous micrometer-sized bubbles [71,72,73,74]. However, these probes are limited by the short life (<30 min) and the micron size of the bubble—hurdles that only nanotechnology can tackle [75,76]. In recent times, Jokerst et al. [77] and others [32,42,43,78,79] have described that nanoparticles formulated using silica can serve as a contrast for US imaging. While the boundary in the middle of the gaseous microbubbles and tissue offer some disparity, the interface in the middle of tissue and the rigid silica particles is also the same [77,80]. Rather than co-injection with microbubbles, the nanoparticles are encapsulated in the cells [65,81] or surface-bound microbubbles [74,82]. This guarantees that the nanoparticle matches the cell of interest. Kempen et al. used Stöber silica nanoparticles in one research, but the probes used had a lengthy biodegradation phase because of their compact silicate system and small dissociation constant of silica; as such, it could not transfer prosurvival mediators to surge the persistence of stem cells. Consequently, another study had to be done employing MSNs [83,84,85,86,87,88], which gave a great surface area (~1000 m^2^/g) [89]. While in the field of cancer, several researchers have used MSNs [90], Kempen et al. [91] theorized that they can serve as prosurvival agents to stem cells and concomitantly as a contrast agent. Another objective they had was to use the high surface area of the MSNs to assist biodegradation and elimination from active beings. In an experiment, they reported the fabrication and authentication of MSNs as a theranostic means that can be very vital in stem cell therapy (Figure 5). An insulin-like growth factor (IGF) was used as a prosurvival model due to its innovative use as an agent in cardiac stem cell therapy (CSCT) [92,93]. The report shows concomitant drug delivery with MSNs as well as ultrasound/magnetic resonance imaging. The ultrasound (US) is beneficial for image-guided delivery and quantifying, while magnetic resonance imaging (MRI) gives an advanced purpose to supplementary studies, and the delivery of drugs averts the death of the stem cell. This method could serve as a key advantage for cardiac stem cell therapy in addition to abdominal uses of reformative treatment pliable to US imaging.

## 5. Brain Cancer Theranostics using Mesoporous Silica Nanomaterials

Brain cancer is well-thought-out as a large unresolved clinical problem, while major progress has been attained in the detection and treatment of other types of cancer. A high-grade malignant glioma, glioblastoma multiforme (GBM), is the most atrocious form of brain cancer characterized by fast growth and damage of the immediate brain parenchyma. High resistance to chemotherapy, intense frequency of relapses, quick neurological damage, as well as very low survival rates classify brain cancer one of the most terrible cancers [94,95,96,97]. Now, available treatment includes chemotherapy or surgical removal of the tumor and, subsequently, radiotherapy or both. Upon all these, the projected average survival of a patient with brain cancer is just 14.6 months. Statistics indicate that less than 5% of patients live more than 5 years. This can be attributed to: (i) The presence of the blood–brain barrier (BBB), which excludes most therapeutic agents; (ii) the infiltrative nature and fast growth of brain tumors ending in incomplete removal; and (iii) tumor recurrence as a result of the development of chemotherapeutic resistance. As such, available conventional chemotherapy is largely ineffective for treating brain cancer. Hence, there is a serious need to devise ways to overcome the boundaries and enhance the efficacy of brain cancer therapies [98,99,100,101]. Nanomedicine offers a promising therapeutic prospect for brain cancer theranostics. The step-by-step progress in nanomaterials has opened countless ways to off-load the several limitations of brain cancer treatment. 

Recently, Mo et al. engineered MSNs with appropriate particle sizes to cross the BBB and antagonize the glioblastoma. They synthesized Crgd peptide conjugate doxorubicin loaded MSNPs of different sizes, i.e., 20, 40, and 80 nm. It was observed that the functionalized nanosystem selectively identifies and attaches to the U87 cells having a high level of ανβ3 integrin, successively causing reticence to glioma cells and enhancing cellular uptake, especially for the particle size of 40 nm. Moreover, doxorubicin-loaded nanomaterials were more selective, and their anticancer action compared to that of free Doxorubicin caused the glioma cells apoptosis by initiating overproduction of reactive oxygen species (ROS). It was detected that doxorubicin-loaded mesoporous silica nanoparticles (MSNPs) with a particle size of 40 nm exhibited stronger penetrability across the blood–brain barrier (BBB) and were capable of disrupting the VM capability of glioma cells by controlling the expression of FAK, E-cadherin, and MMP-2, and as a result achieving reasonable anti-glioblastoma efficacy and preventing the undesirable toxic adverse effects on viable brain tissue. This outcome indicates that reducing or adjusting the particle size of MSNs’ nanosystem can be a way to alienate glioblastoma and cross the BBB [102]. In a new study, Liu et al. designed three different sizes of PEGylated SiNPs (PSiNPs) to cross the BBB and performed in vivo and in vitro analysis. In vivo analysis entailed animal experiments, superficial in vivo imaging, and ex vivo optical imaging after injection through the carotid artery [103].

In an innovative study, Bertucci et al. synthesized nanomaterials of 100 nm in size, further integrating a Cy5 fluorophore within the silica framework. This was then loaded with anticancer drug temozolomide (TMZ)—for treating gliomas. The particles surface was then coated with a poly-arginine–peptide nucleic acid (R8-PNA) conjugate targeting the miR221 microRNA. The results indicated that the multifunctional nanosystem is swiftly internalized into glioma C6 or T98G cells. The anti-miR action of the PNA was retained. In addition, induction of apoptosis was observed in temozolomide resistant cell lines, as this was confirmed by reverse transcription polymerase chain reaction (RTPCR) measurements. The TMZ-loaded mesoporous silica nanomaterial showed an enhanced pro-apoptotic effect, and effective induction of apoptosis (70.9% of apoptotic cells) resulted from the additive effect of TMZ and R8-PNA in the mesoporous silica nanomaterials thus far achieved in the temozolomide-resistant T98G cell line [104]. 

## 6. Mesoporous Silica Nanomaterials for Plant Gene Delivery

**Figure 6:**
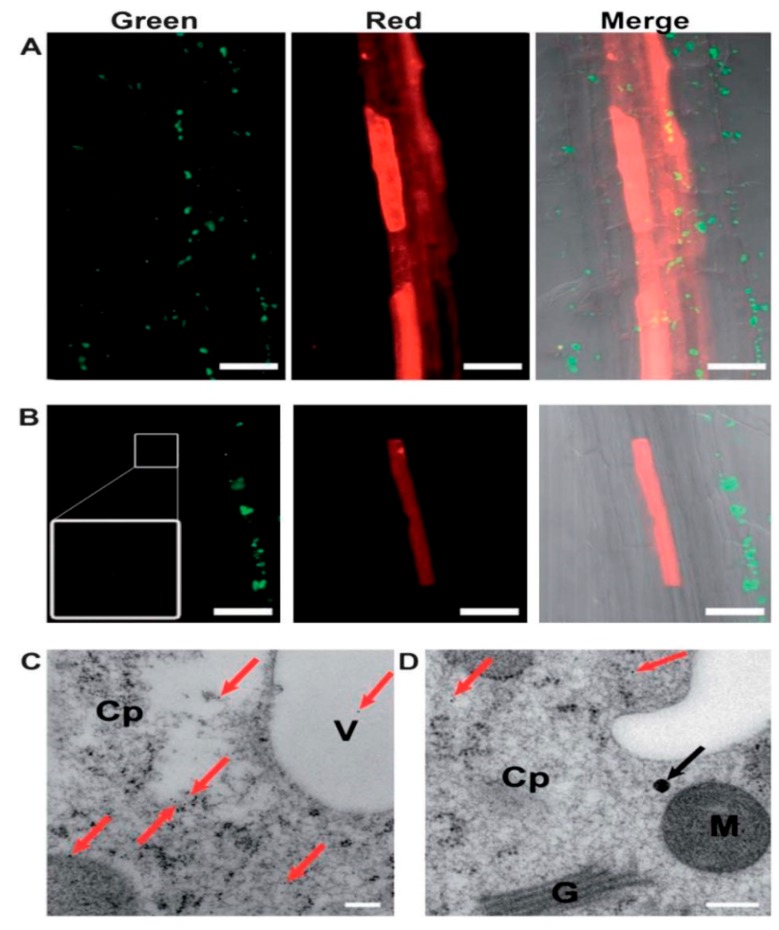
Delivery of genes (MSN-mediated). (**A**,**B**) Confocal microscopy of Arabidopsis root cells treated with DNA–MSN complexes (1:100 ratio) for 48 h at 24 °C in 1/2 MS. Gene expression (mCherry protein; red) was observed in endodermal (**A**) and cortical (**B**) cells. TMAPS/F-MSNs were present in cells expressing mCherry (**B**, green channel). Scale bars: 50 mm. (**C**,**D**) TEM of immunogold-labelled mCherry protein in root cells after incubation with DNA–MSN complexes. Red arrows show the gold-labeled mCherry proteins. Presence of TMAPS/F-MSNs (black arrow) and mCherry protein (red arrows) in the same cell (**D**). Scale bars are 200 nm. Cp, cytoplasm; M, mitochondrion; V, vacuole; G, Golgi apparatus. Reproduced from Reference [120] with permission from The Royal Society of Chemistry.

Applications of mesoporous nanomaterials (MSNs) have been established in many biomedical areas, such as cell pointers for bioimaging (fluorescence and MRI) [105,106], stimuli-responsive drug delivery [107,108,109], protein and enzyme delivery [84,110], DNA or RNA transfection [111,112,113], and multipurpose theranostic agents [61,114]. In mammalian systems, the reaction of markers of biological origin to MSNs, comprising its biodegradability, in vivo cytotoxicity, and biocompatibility, has been observed [115,116]. Nonetheless, there is a big difference in plant cell morphology, unlike in mammalian cells. Now, several research works are geared towards the phytotoxicity of nanoparticles [117,118,119] as well as the impact of these nanomaterials on the growth of plants [120,121,122]. In spite of little research in plant sciences, reports indicate that nanoparticles can be used in plant cells, among those being anatase TiO_2_–alizarin red S nanoconjugates [123], single and multiwalled carbon nanotubes [120,124,125], quantum dots (CdSe/ZnS) [126], and magnetic nanoparticles (carbon-coated) [127,128]. The minute nanoparticles permeate the plant cells in several ways, such as creating new pores and attaching to carrier proteins and ion channels. [129]. Few research works suggest that nanoparticles can be used as drug delivery agents [130]. There are few other research works illustrating the delivery of biomolecules into plants employing nanoparticles [83,131,132,133,134,135,136,137]. Hitherto, a lot of these studies used protoplasts [83,133] or cells [133,135,136] as targets without taking into consideration differentiated tissues. Protoplasts are plant cells that do not have a cell wall and, hence, macromolecules like nanoparticles could be co-opted by the process of endocytosis. Existing nanoparticle-mediated techniques capable of delivering biomolecules into plants cell walls are primarily based on ultrasound or rely on mechanical forces [83,131,132] to enter the cell wall [135,138]. An ultrasonic method which is used on culture cells is inexpensive and easy to use but cannot be used in this case. Of late, gold-loaded mesoporous nanomaterials (MSNs) have been established to convey protein, DNA, and compounds via the gene gun technique [83,131]. However, the carriers with the biomolecules only target the plant tissue surface. Low endocytosis activity and the cell wall barrier of plant cells limit the application of nanotechnology on plant systems. Chang et al. in 2013, successfully showed, with a simple co-culture method, MSNs can penetrate the cell wall and be used a carrier in Arabidopsis roots (Figure 6) [120]. A merit is that the carriers can penetrate the underlying tissues and innumerable vital organelles. The modern technique possesses a number of advantages: (i) MSNs convey DNA to deeper tissues, such as the endodermis and cortex; (ii) for degrading the plant cell walls, no enzymatic treatment is required; (iii) a very scanty dose of DNA (0.2 mg per treatment) is needed to get a practical efficiency for transient gene expression; (iv) a promising direction for biomolecule delivery because of association of an energy-independent route for MSN uptake; (v) MSNs are capable of moving to a number of organelles, for instance, nuclei and plastids, in order to be used in targeted delivery, for example, plastid transformation; (vi) MSNs possessing nanopores are promising as multipurpose vehicles for transporting several agents into cells in undamaged plants.

## 7. Intracellular Delivery of Nucleic Acids via Mesoporous Silica Nanomaterials

**Figure 7:**
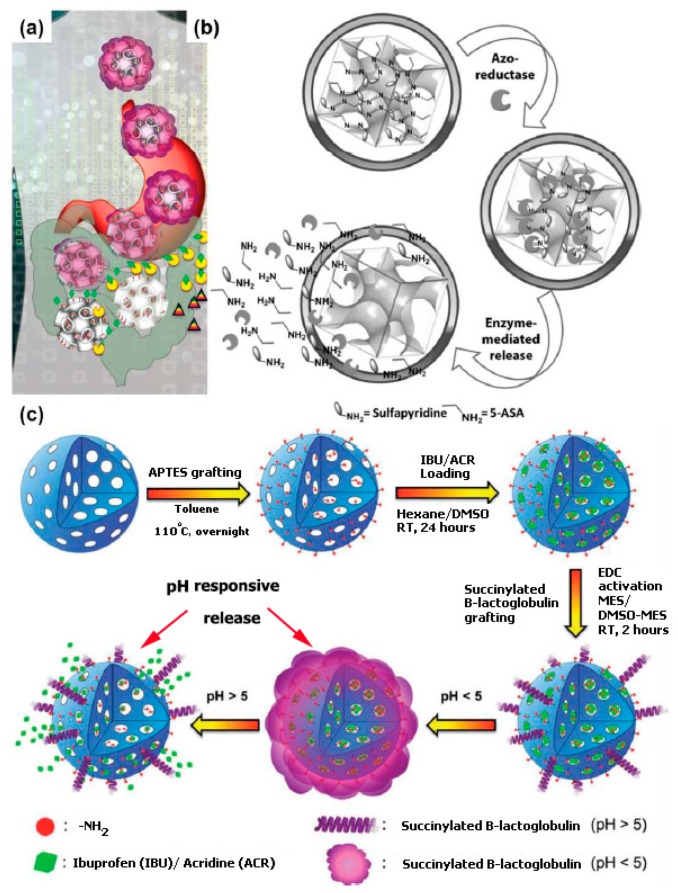
(**a**) Schematic representation of targeted drug delivery to the lower part of the gastrointestinal (GI) tract. Reproduced from Reference [145] with permission from Elsevier Ltd.; (**b**) delivery system with anti-inflammatory azo-prodrug (sulfasalazine). Reproduced from Reference [146] with permission from John Wiley & Sons, Inc.; (**c**) pH-sensitive nanocarrier obtained from MSNs modified with ß-lactoglobulin. Reproduced from Reference [147] with permission from John Wiley & Sons, Inc.

To date, the most successful and efficient method for gene therapy has been virus-mediated gene delivery [139]. Yet serious safety concerns associated with this method are a challenge. For some years now, mesoporous silica nanoparticles (MSNs) have gained much consideration for intracellular delivery of nucleic acids. The delivery of cellular plasmid DNA (pDNA) is engineered to replace and restore the function of a defective gene to its normal state in the cell [140,141]. Transfer of small interfering RNAs (siRNAs) can selectively knock down genes by specifically targeting mRNAs. The biocompatibility and distinctive structures of MSNs make it an ideal candidate to act as a biomolecule carrier. Gene delivery introduces remote nucleic acids into target cells exclusively for therapy. pDNAs bearing vital genes to rectify the function of a target gene (e.g., an oncogenic gene) have been employed for gene therapy to treat specific ailments. Small interfering RNAs (siRNAs) are striking as anticancer therapies as they can alter expression of cell cycle regulators, activated oncogenes, or proapoptotic genes, all hypothetically critical for tumor survival or tumorigenesis [142,143,144]. A number of research studies have described attempts to load nucleic acids into MSN preparations for delivery into cells (Figure 7).

### 7.1. pDNA Delivery

Using a strong constitutive promoter to replace the defective gene, intracellular delivery of pDNA enhances the expression of therapeutic genes and restores normal function. Some studies have investigated the possibility of using monodispersed MSNs loaded with pDNA as a carrier into cells (Table 2). Research by Radu et al. [111] proved that a polyamidoamine (PAMAM) dendrimer coating produced a positively charged surface capable of complexing to DNA while allowing the pores to encapsulate drugs and dyes. This study also showed that MSNs protected the DNA against enzymatic cleavage and that they were not toxic. Other polymers, such as poly (allylamine hydrochloride) (PAH) [148] and polyethyleneimine (PEI) [149], are also excellent coatings to enhance complexation of DNA on the MSN surface. Both cationic polymeric coatings boosted DNA loading onto MSNs and transfection efficiency.

### 7.2. siRNA Delivery 

siRNAs can specifically knock down target genes by targeting specific mRNAs. Various studies of siRNA delivery with MSNs employed cationic polymeric coatings, like PEI, comparable to those used in DNA delivery studies (Table 2). Hom et al. [154] reported that siRNA was attached to the positively charged PEI external surface of MSNs through electrostatic force. This study they performed also showed that the PEI coating stimulated endosomal escape of the siRNA into the cytosol, which is crucial for ample effective target gene knockdown efficiency, in pancreatic cancer cells. Other researchers also worked on PEI-coated MSNs for siRNA delivery into breast cancer cells [165]. Due to intracellular siRNA mediated knockdown of the target, TWIST 1, a transcription factor regulating angiogenesis, they detected reduced tumorigenic activity. PEI can similarly be functionalized with new molecules before attachment to MSNs, including cyclodextrin [161] or acetaldehyde–cysteine (AC) [162]. In a current study by Möller et al., exceptionally high siRNA loadings were observed when even medium-sized pores (4 nm) with highly positively charged internal pore surfaces were used [167]. A cationic amphipathic cell-penetrating peptide, KALA, which is also a targeting peptide when added to the MSN surface, produced enhanced endosomolytic function. Chen et al. [163] and Li et al. [158] engineered MSNs coated with PEI and afterwards conjugated to KALA peptides. Although the MSN pores (<5 nm, comparable to siRNA size) were small, the highly positive surface charges (+23.6 mV [48] and +25 mV [43]) permitted siRNA complexation and led to excellent delivery into target cells, with effective tumor inhibition and gene silencing. PEGylated liposomes have also been employed as coating agents of MSNs after encapsulating siRNA in the pores (23–30 nm), generating excellent transfection efficiency in Hep3B cells [112]. MSNs were synthesized with magnetic cores, forming yolk–shell or core–shell structures. The magnetic cores allowed tailored delivery of siRNAs to target sites channeled by an external magnetic field. A number of studies reported that by synthesizing magnetic iron oxide cores via mesoporous silica shell coated with PEI, efficient endosomal escape and effective intracellular siRNA delivery was achieved in A549 [159], HeLa [156], and KHOS [164] cells. All these examples prove that positive charge is essential for gene complexation, but further studies are required on optimal pore size.

### 7.3. Co-Delivery of Nucleic Acids and Drugs

Over the past few years, significant advances have been achieved using MSNPs as combinatorial delivery systems. Through MSNs, drugs have been delivered together with nucleic acids to maximize delivery of the drug into the cell, as well as to enhance the efficacy of the drug being delivered (Table 3). The old method of gene/drug delivery encompassed injection of a gene carrying virus together with administration of a therapeutic drug straight into the tumor. Nonetheless, owing to differences in the pharmacokinetics of the drug and the nucleic acid, this usually led to low drug efficacy. MSNs are highly useful for such co-delivery systems because of their well-established surface chemistry enabling surface modifications, large surface area, and porous structures supporting encapsulation of both nucleic acids and drugs [168]. Chen et al. described an early example of co-delivery of a drug/nucleic acid combination [169]. They evaluated the delivery of a siRNA targeted at Bcl-2, a gene regulating apoptotic cell death along with doxorubicin, an apoptosis-inducing agent, into A2780/AD ovarian cancer cells. Bcl-2 siRNA was attached to PAMAM-coated MSNs through the electrostatic bond between the positively charged dendrimer and the negatively charged RNA, whereas doxorubicin was loaded into the stomata of the mesoporous silica nanomaterials (MSNs). After the co-delivery system was tested in cells, it was observed that doxorubicin co-delivered with siRNA was more cytotoxic than that delivered without siRNA.

Comparable observations were made in other cell types including A549 [170], HeLa [171,172], and MDA-MB-231 [173] cells, also by means of Bcl-2-targeted siRNA and doxorubicin in combination, albeit with trivial alterations to coatings/polymer. Additionally, Taratula et al. [170] showed in an in vivo biodistribution experiment that the MSNs, when inhaled, were localized and confined in the lungs of mice. Further siRNA types were also tested in combination with doxorubicin [174]. Doxorubicin with P-glycoprotein (Pgp) siRNA was used to combat multidrug resistance in two types of cancer cells, MCF-7 [175] and KB-V1 [174]. In both studies, PEI was used as the cationic polymer coating for siRNA complexation, whereas phosphonate was used to coat the pores to electrostatically bind doxorubicin within them. These nanomaterials produced efficient intracellular co-delivery of siRNA and doxorubicin. Considering previous instances of systems transporting nucleic acids only, investigations were done for other coatings using combined delivery systems. Kar et al. [176] attached poly-L-arginine to the surface of MSNs to complex pDNA, with doxorubicin in the pores. Poly-l-arginine does not just impart a positive charge to complex DNA, but MSNs synthesized this way also exhibited excellent cell penetration in both A549 and HeLa cells. At large, these studies indicated that the genetic material complexed to the surface of the particle through positively charged groups/polymers, and the drugs are encapsulated in the pores (which were small, <5 nm). Modern research progresses make nucleic acid delivery into cells using MSNs a conceivable prospect. 

## 8. Oral Drug Delivery Using Mesoporous Silica Nanomaterials 

Presently, a further effective oral delivery of drugs and therapeutic agents through the gastrointestinal (GI) tract is of great interest, as there is a surge in the development of new platforms. Considering the oral delivery method, drugs with good bioavailability can easily reach systemic blood circulation via the mouth; thus, this method appears as one of the simplest and most patient-friendly methods to self-administer drugs and ensures great compliance with the patient. The rate of oral absorption is controlled by the physiology of the patient. However, intestinal mucus and barriers present in the gastrointestinal tract ensure efficient systematic absorption of a drug if it is administered via the buccal cavity. Hitherto, efficient delivery of poorly water-soluble drugs with low metabolic stability and/or low permeability and making effective formulations are still very a big hurdle for the pharmaceutical industry. Moreover, conventional oral drug delivery methods are laden by a number of factors. To curtail these challenges, diverse formulation approaches and delivery pathways have been studied [13,177,178,179,180,181,182,183,184,185]. Though a few of the old drug carriers have successfully solved the problems related to the crystallization issues or delivery of poorly soluble drugs, only a handful of them attained positive results. These problems have made the scientific world think through other approaches toward targeted drug delivery systems in order to get smart drug delivery carriers. 

Despite the several advantages of the oral administration route, oral delivery of peptides and/or protein drugs is limited. This method is limited because proteins (peptides) are prone to enzymatic and chemical degradation in the gastrointestinal tract. Thus, with regard to oral delivery of protein and peptide drugs, the use of protecting coatings and nanocapsules has become necessary to fully protect the active compound from the GI environment (Figure 7). Foremost, tackling the issue of premature release of bioactive complexes loaded in the nanocarriers is very serious mainly with drugs that are expensive. Kleitz and Qiao [146] labeled a carrier in which an anti-inflammatory azo-pro-drug (sulfasalazine, SZ) was covalently bound to MCM-48-type (3-D cubic pore structure) nanoparticles, and this scheme acts as an enzyme responsive carrier. The reduction of the azo-bond can be triggered by the colon microflora azoreductase, and this activates the drug release (Figure 7).

Amid diverse modification processes, principally moving are schemes based on the use of biodegradable nutraceuticals, such as soy protein isolates (SPI) [186], β-lactoglobulin [147] or chitosan [145,187]. β-lactoglobulin is a whey protein, present in sheep and cow milk, and it is fully biodegradable and biocompatible. Conversely, chitosan is an FDA-approved, naturally occurring biodegradable polysaccharide with a wide array of applications in drug delivery and wound healing [188,189,190]. β-lactoglobulin- and Chitosan-modified materials can be used as effective pH-responsive drug delivery supports which allow for tailored drug release in different pHs (Figure 7). For example, Guillet-Nicolas et al. used the benefits of MSNs with the advantages of the reasonably priced nutraceutical β-lactoglobulin, which is also a surplus product of the dairy industry. In this method, MSNs (MCM-48-type nanoparticles) functionalized with aminosilane were linked to succinylated β-lactoglobulin (in the presence of 1-ethyl-3-(3-dimethylaminopropyl) carbodiimide) and loaded with a drug molecule (ibuprofen, as a model drug) [147]. The resulting bioconjugates were tested as a novel pH-responsive oral drug delivery system. This pH-responsive gating device exhibited low toxicity and exceptional colloidal in simulated intestine fluid (SIF). Most significantly, this pH-sensitive carrier displayed sustained drug release in intestinal physiological conditions (pH = 7.4) and narrow untimely release was detected in acidic media (replicated gastric fluid, SGF, pH 1.2). Such a controlled release profile was credited to the major conformation modifications of the protein in low pH where ß lactoglobulin undergoes a gelation process, forming a pH-dependent shell (gel) covering the nanoparticles. By contrast, higher pH (6.8–7.5) led to the reverse effect, permitting the active substance to diffuse out. Popat et al., in comparison, have covalently bonded positively charged chitosan polymer to phosphonate-functionalized MCM-41 nanoparticles [145,187]. Their results showed a little different release profile, i.e., around 90% of ibuprofen was released in pH = 5, whereas only 20% of the drug was released in the colon environment (pH = 7.4). By contrast, uncoated silica particles exhibited a fast release of ibuprofen in the two values of pH tried. 

## 9. Conclusions and Future Perspective

In this review, mesoporous silica nanoparticles can be thought of as using one stone to kill three birds, i.e., in cancer theranostics, gene delivery, and drug delivery. As it stands, there are several ways to deliver drugs to their targeted site of action. The drugs can be either directly loaded into the nanoparticles or indirectly designed as a prodrug that is dormant until they fuse with specific enzymes or molecules. For the preparation of a versatile system to ensure drug delivery, MSNs are in the domain as candidates. MSNs have also elicited immense advantages for the delivery of anticancer agents over other nanocarriers with respect to cancer therapy, such as excellent drug loading capacity and endocytotic nature. The outer shells of mesoporous nanomaterials can further undergo modifications using countless stimuli-responsive and tumor-recognition molecules that will boost the therapeutic effect of anticancer agents. Additionally, the capacity of mesoporous nanomaterials to undergo energy-independent endocytosis and co-delivery can overcome the multidrug resistance in cancer cells. 

To conclude, the advancement of MSN-based drug delivery is a great tool for cancer theranostics and drug and gene delivery. Considering the properties and versatility of MSNs, they are well thought out as a perfect drug delivery vehicle or system. Furthermore, MSN-based drug delivery vehicles or systems can undergo conjugation with multipurpose molecules, which can be employed in diverse delivery platforms, including optical imaging, a controlled-release process for the management of ailments and drug delivery. With all that is being done to overcome the setbacks in cancer therapy and drug and gene delivery, it is useful to foresee a definite multifunctional MSN-based drug delivery system as promising to catalyze the evolvement of drug and gene delivery and cancer theranostics. 

## Figures and Tables

**Table 1:** Cancer therapy using silica nanomaterials.

Initial Cancer Detection and Diagnosis	Drug Delivery Systems Used	Anti-MDR MSN-Based Systems	Reference
Imaging Contrast Agents	Utilizes the Passive delivery system	Concurrent delivery of multiple drugs	[32,33]
Mesoporous Silica Nano Chips	Utilizes the Active delivery system	Co-delivery of genes and anticancer drugs	[34]
Optical Imaging using Fluorescent Silica Nanoparticles	Utilizes the Controlled-release drug delivery systems	Multiple combination delivery systems	[35]

**Table 2:** Particle and pore sizes and surface coatings in nucleic acid delivery systems with mesoporous silica nanoparticles (MSNPs).

Nucleic Acid	Surface Coating	Size, nm	Cell Type Tested	Zeta Potential, mV	Reference
pDNA	PAMAM	particle: 250; pore: 2.7	HeLa CHO	–	[111]
	PAH	particle: 150; pore: 9.8	PC1	+20	[148]
	mannosylated PEI	particle: 60–130; pore: not measured	RAW 264.7 HeLa	+5 to +50	[149]
	aminopropyl groups	particle: 70–300; pore: 20	–	–	[150]
	amino-functionalized MSN	particle: 300–400; large pore: 23; small pore: 2	HeLa	+10 to +20	[151]
	amine groups	particle: 205; pore: 2.6	MSC PC12 HeLa CHO	+23	[141]
	lipid bilayer	particle: 230; pore: 2-5	RAW 264.7 HEK293	+24	[152]
	APTES and l-histidine	particle: 100-180; pore: 2.5	HEK293T7	+5	[153]
siRNA	PEI	particle: 100; pore: 2.5	PANC-1	–	[154]
	PEGylated liposomes	particle: 165; pore: 23–30	Hep3B	+12	[112]
	APTES and PEG	particle: 200; pore: 23	HeLa	+8	[155]
	PEI	particle 63; pore: not measured	HeLa	+48	[156]
	Poly-l-lysine (and APTES)	particle: 100-200; pore: 28	HeLa KHOS	PLL +2 APTES +3	[157]
	Fe_3_O_4_ core with mesoporous silica shell, and coated with PEI	particle: 50; pore: 3.6	A549	+25	[158]
	PEI	particle: 50; pore: 3.7	A549	–	[159]
	PDMAEMA	particle: 100–150; pore: 10	HeLa-Luc	+27	[160]
	cyclodextrin and PEI-functionalized MSN	particle: 105; pore: not measured	MDA-MB231	+47	[161]
	PEI	particle: 178 (without siRNA); pore: 19	KHOS	+34	[162]
	PEI, PEG and KALA peptide	particle: 50; pore: 2.6	A549 L02 PC-3 HCCLM-3	+23	[163]
	PEI	particle: 200; pore: 11	KHOS	+51	[164]
	PEI	particle: 127; pore: 2–3	MDA-MB435S	+44	[165]
	APTES + tannic acid	particle: 150; pore: 12	KHOS	+21	[166]
	cationic block copolymer	particle: 150; pore: 4	KB	+30 to +40	[167]

**Table 3:** Particle and pore sizes and surface coatings in systems for co-delivery of drugs with nucleic acids using MSNs.

Surface Coating	Size, nm	Zeta Potential, mV	Nucleic Acid	Co-Delivered Drug	Cell Type Tested	Reference
PAMAM	particle: 200 pore: 2.88	–	siRNA	DOX	A2780/AD	[169]
PEI	particle: 100–120 pore: 2–2.5	+30 to +35	siRNA	DOX	KB-V1	[174]
PEG-LHRH peptide (luteinizing hormone-releasing hormone)	particle: 160–180 pore: 2.83–2.99	–	siRNA	DOX CDDP	A549	[170]
Poly-**l**-arginine-grafted MSNs	particle: 35–60 pore: 2.6	+20 to +32	pDNA	DOX	HeLa A549	[176]
PEI-PEG copolymer	particle: 50 pore: not measured	+25	siRNA	DOX	MCF-7 (multi-drug resistant)	[175]
PEI conjugated to folic acid	particle: 185 pore: 2.6	+28	siRNA	DOX	HeLa (high folic acid expression) MCF-7	[172]
Ethylenediamine-modified β-cyclodextrin	particle: 150 pore: 2.7	+20	siRNA	DOX	HeLa	[171]
PEI-PLL copolymer	particle: 249 pore: 2.6	+26	siRNA	DOX	MDA-MB-231	[173]
